# Supplementary material for: An Evidence-Based Somatic Acupressure Intervention Protocol for Managing the Breast Cancer Fatigue-Sleep Disturbance-Depression Symptom Cluster: Development and Validation following the Medical Research Council Framework
Source: Int J Environ Res Public Health. 2022 Sep 21;19(19):11934. doi: 10.3390/ijerph191911934 (PMC9565572; doi:10.3390/ijerph191911934)
Supplement: Supplementary file 1 [file ijerph-19-11934-s001.zip › ijerph-1886199-supplementary.pdf]

Supplementary File: Indications, effects and roles of the selected acupoints

Acupoints	Meridian	Location	Indications, effects and roles
Zusanli (ST36)	Stomach Meridian	3 cun (four finger breadths) below the kneecap, and one thumb breadth from the lateral of the tibia's anterior crest	▪ Tonify *qi* and blood (1)<br>▪ Commonly used for managing fatigue, insomnia and depression (2)(3)(4)
Sanyinjiao (SP6)	Spleen Meridian	Medially 3 cun (four finger breadths) above the ankle (inside)	▪ Invigorate the spleen to reinforce *qi* and nourish the liver to tonify *yin* and blood (5) (6)<br>▪ Commonly used for managing fatigue, insomnia, palpitations and anxiety (2)(3)(4)
Taixi (KI3)	Kidney Meridian	In the depression between the medial malleolus tip and calcaneal tendon	▪ Tonify kidney *qi* and nourish kidney *yin* (7)<br>▪ Commonly used for managing fatigue and insomnia (2)(3)(4)
Hegu (LI4)	Large Intestine Meridian	Between the first and second metacarpals, at the midpoint of the radial side of the second metacarpal	▪ Promote the circulation of *qi* and blood (8)<br>▪ Commonly used for symptoms in relation to *qi* and blood stagnation such as fatigue, insomnia and depression (2)(3)(4)
Neiguan (PC6)	Pericardium Meridian	Between the palmaris longus tendons and flexor carpi radialis muscles, and 2 cun above the wrist crease	▪ Promote the circulation of *qi* and blood (9)<br>▪ Commonly used for symptoms from *qi* stagnation such as insomnia and spirit disorders (2)(3)(4)
Shenmen (HT7)	Heart Meridian	In the depression radial to the proximal border of the pisiform bone	▪ Tonify deficiencies of the heart *qi*, calm the heart and tranquilize the mind (10)<br>▪ Commonly used for managing insomnia and emotional distress (2)(3)(4)
Baihui (GV20)	Governing Vessel Meridian	The intersection of the median line of the head and the line connecting the highest points of two ears	▪ Soothe the liver, rectify *qi*, calm the heart and the mind (11)<br>▪ Commonly used for symptoms related to liver function depression and *qi* stagnation, and is recommended for managing depression and insomnia (2)(3)(4)
Qihai (CV6)<br>Guanyuan (CV4)	Conception Vessel Meridian	On the anterior median line and 1.5 cun below the umbilicus	▪ Build and store *qi* via complementing the congenital deficiency, and tonify kidney and consolidating the root (12)<br>▪ Commonly used for symptoms related to *qi* deficiencies such as fatigue (3)(4)
Yintang (EX-HN3)	Extra point	The midpoint between the medial ends of the two eyebrows	▪ Soothe the liver, rectify *qi*, calm the heart and the mind (11)<br>▪ Commonly used for symptoms related to liver depression and *qi* stagnation, a recommended acupoint for managing depression and insomnia (2)(3)(4)

Taichong (LR3)	Liver Meridian	On the foot dorsum, in the depression distal at the junction of the first and second metatarsal bone	▪ Promote the flow of *qi* and blood in the body [8]<br>▪ Commonly used for insomnia [2][3][4]

(1) Grant SJ, Smith CA, De Silva N, et al. Defining the quality of acupuncture: the case of acupuncture for cancer-related fatigue. Integrative Cancer Therapies. 2015 May;14(3):258-70. (2) Liu XL, Cheng HL, Moss S, Wang CC, Turner C, Tan JY. Somatic Acupoint Stimulation for Cancer-Related Sleep Disturbance: A Systematic Review of Randomized Controlled Trials. Evidence-Based Complementary and Alternative Medicine. 2020 Apr 29;2020. (3) Liang FR, & Wang H. Acupuncture and moxibustion (4th edition). Beijing: China Press of Traditional Chinese Medicine Co.Ltd. 2016. (In Chinese). (4) Shen XY. Meridians and acupoints (4th edition). Beijing: China Press of Traditional Chinese Medicine Co.Ltd. 2008. (In Chinese). (5) Zhang Z & Zhang P. Curative Observation on Needling Neiguan (PC6), Zusanli (ST36), and Sanyinjiao (SP6) to Treat Insomnia. International Journal of Clinical Acupuncture. 2014 Oct 1;23(4). (6) Mallory MJ, Croghan KA, Sandhu NP, et al. Acupuncture in the postoperative setting for breast cancer patients: a feasibility study. The American journal of Chinese medicine. 2015 Feb 10;43(01):45-56. (7) Han X, Fan Y, Zhao H, et al., Acupoint Selection Rule Mining of Premature Ovarian Failure Treatment with Acupuncture and Moxibustion Based on the Data Analysis of Clinical Literature. In2018 IEEE International Conference on Bioinformatics and Biomedicine (BIBM) 2018 Dec 3 (pp. 1866-1871). IEEE. (8) Lai XS, Mo FZ, Jiang GH, et al. Observation on clinical effect of acupuncture on superoxide dismutase, lipid peroxide and nitric oxide in vascular dementia patients. Chinese Journal of Integrated Traditional and Western Medicine. 1999 Dec 1;5(4):269-74. (9) Lin M, Fan L, Liu X, et al. Clinical study on treatment of neck type cervical spondylosis by Acupoints of Neiguan (PC6) combined with medius haemospasia. In2014 IEEE International Conference on Bioinformatics and Biomedicine (BIBM) 2014 Nov 2 (pp. 91-94). IEEE. (10) Panthi S & Gao T. Diagnosis and management of primary hypothyroidism in Traditional Chinese medicine (TCM) and Traditional Indian Medicine (Ayurveda). Int J Clin Endocrinol Metab 1 (1): 009. 2015;12(009). (11) Yu FE, Wang XY, Li SD, et al. Clinical research of acupuncture on malignant tumor patients for improving depression and sleep quality. Journal of Traditional Chinese Medicine. 2011 Sep 1;31(3):199-202. (12) Lin CH, Zhao XY, Liu X, et al. Observation on the mechanism of acupuncture treatment for generalized anxiety disorder using Lieque (LU7), Zhaohai (KI6) as the main acupoints. In2013 IEEE International Conference on Bioinformatics and Biomedicine 2013 Dec 18 (pp. 134-137). IEEE.
